# Risk Factors of Angiographic Recurrence After Endovascular Coil Embolization of Intracranial Saccular Aneurysms: A Retrospective Study Using a Multicenter Database

**DOI:** 10.3389/fneur.2020.01026

**Published:** 2020-09-15

**Authors:** Zhongbin Tian, Jian Liu, Ying Zhang, Yisen Zhang, Xiaolong Zhang, Hongqi Zhang, Ming Yang, Xinjian Yang, Kun Wang

**Affiliations:** ^1^Department of Interventional Neuroradiology, Beijing Neurosurgical Institute and Beijing Tiantan Hospital, Capital Medical University, Beijing, China; ^2^Department of Radiology, Huashan Hospital, Fudan University, Shanghai, China; ^3^Department of Neurosurgery, Xuan Wu Hospital, Capital Medical University, Beijing, China; ^4^Department of Neurosurgery, Wuhan General Hospital of Guangzhou Military Command, Southern Medical University, Wuhan, China

**Keywords:** multicenter, recurrence, packing density, endovascular treatment (EVT), intracranial aneurysm

## Abstract

**Background:** Endovascular therapy of intracranial aneurysms has a high recurrence rate. This study aimed to evaluate the risk factors of recurrence after endovascular coil embolization of intracranial aneurysms.

**Methods:** From January 2014 to May 2015, 504 patients with 558 intracranial aneurysms who were treated by endovascular therapy were recruited from four high-volume centers. We used multivariate Cox proportional hazard regression to evaluate the risk factors associated with the angiographic recurrence of intracranial saccular aneurysms after endovascular coil embolization.

**Results:** Angiographic follow-up was available for 504 patients (558 aneurysms), with a mean duration of 11.42 months. Of the 558 aneurysms, 57 (10.2%) aneurysms showed recurrence. Aneurysm size (*p* = 0.028), therapy (non-stent assisted coiling or stent-assisted coiling) (*p* = 0.008), the Raymond scale (*p* = 0.040), aneurysm rupture status (*p* < 0.001), and packing density (*p* < 0.001) showed significant associations with angiographic follow-up outcome. A low packing density was independently associated with aneurysmal recurrence after multivariate Cox proportional hazard regression analysis (*p* < 0.001).

**Conclusion:** Endovascular treatment is effective for these lesions. Multiple factors could attribute to the aneurysmal recurrence after endovascular coil embolization. The low packing density is the independent risk factor for aneurysmal recurrence. These findings should be verified by larger multicenter and multi-population studies.

## Introduction

Endovascular treatment of intracranial aneurysms (IAs) is an effective and safe alternative to neurosurgical treatment (1). A disadvantage of endovascular therapy of IAs is the higher recurrence rate compared with surgical clipping. The rate of aneurysmal recurrence ranges from 6.1 to 33.6% (2–4). Several risk factors related to recurrence of aneurysms have been reported, such as the rupture status, the size of the aneurysm, and the state of with or without stents (5–8). However, many studies obtained their conclusions based on single-center data or limited numbers. In this study, we retrospectively analyzed the data of 504 patients with 558 IAs from four high-volume neurointerventional centers. All of these patients had angiographic follow-up results. This study aimed to evaluate the risk factors of recurrence after endovascular coil embolization of intracranial aneurysms.

## Materials and Methods

This study was approved by the ethics committee of four institutes. Informed consent from all of the patients or their relatives was obtained during hospitalization.

### Selection of Patients and Demographics

From January 2014 to May 2015, we retrospectively collected data on patients with intracranial saccular aneurysms who were treated by endovascular coil embolization with or without the stent(s). Some patients were excluded based on the following criteria: absence of angiographic follow-up, existence of mycotic, dissecting, obliterative or inflammatory aneurysms, those who were previously clipped surgically, and those with cerebral arteriovenous malformations or fistulas. A total of 504 patients with 558 intracranial saccular aneurysms who had angiographic follow-up were enrolled in the present study. We divided them into two groups according to angiographic follow-up results. Group I included the aneurysm with contrast filling kept stable or decreased in the follow up compared with the immediate angiographic outcome after the endovascular procedure. Group II contained the aneurysms that were considered as recurrence defined as any increase in contrast filling of the aneurysms during follow-up compared with the immediate angiographic outcome after the endovascular procedure (9). The retreatment decision was based on evaluation of recurrence by an independent radiographic committee. In cases where residual aneurysms were >20% of the original lesion, unstable neck remnants and aneurysmal regrowth were considered to be treated. The locations of aneurysms were described as two subgroups according to the parent artery. One subgroup was the anterior circulation, which consisted of the anterior communicating artery, anterior cerebral artery, internal carotid artery, and middle cerebral artery. The other subgroup was the posterior circulation arteries, which consisted of the posterior cerebral artery, vertebral artery, basilar artery, superior cerebellar artery, anterior inferior cerebellar artery, and posterior inferior cerebral artery. The angiographic results were evaluated by an independent radiographic committee that consisted of five neurointerventionalists with more than 10 years of experience who were not involved in this study. Data of the patients' demographics (age and sex), aneurysm size, aneurysm location, aneurysm shape (an irregular shape indicated an aneurysm with blebs, nipples, or multiple lobes), aneurysm rupture status identified by head computed tomography and/or intraoperative findings, packing density (the ratio between the volume of the coils and the volume of the aneurysms) (10), potential risk factors (hypertension, diabetes, hyperlipemia and smoking), endovascular treatment therapy (non-stent assisted coiling or stent-assisted coiling), treatment complications, and angiographic and clinical follow-up outcomes were collected. We then statistically analyzed the data.

### Interventional Technology and Medical Therapy

All of the patients were treated via the endovascular approach. Generally, the stent would be applied if the aneurysm neck was wide (≥4 mm) or the dome-neck ratio (≤2) was unfavorable. All of the procedures were performed under general anesthesia and systemic heparinization. If a stent was used in patients with remote subarachnoid hemorrhage, dual antiplatelet medication (100 mg aspirin and 75 mg clopidogrel per day) was administrated for 3 days before the procedure. If the aneurysm was acutely ruptured, patients who were clearly conscious were administered a loading dose of 300 mg aspirin and 300 mg clopidogrel either orally or rectally, 2 h before the scheduled stenting. Unconscious patients received a loading dose of tirofiban (intravenously at a rate of 8.0 mg/kg over 3 min) after the stent successfully reached the designated segment of the parent artery. Then tirofiban was given at a rate of 0.1 mg/kg/min for 24 h. An unruptured aneurysm was also administrated dual antiplatelets for 3 days if a stent was used. Systemic heparinization was started after introducing the first coil and before placement of the stent. After the procedure, 75 mg clopidogrel was administered each day for 6 weeks and 100 mg aspirin was administered each day for 6 months. Before each coil detachment and at the end of the procedure, digital subtraction angiography was performed to assess the patency of the parent vessel and to ensure that all coils were confined to the target aneurysm.

### Angiographic and Clinical Follow-Up

Angiographic occlusion results were evaluated and classified according to the Raymond scale, as described above (9). Complete embolization (grade 1) showed no contrast filling of the dome, body, or neck of the aneurysm. A neck remnant (grade 2) was defined by a residual neck (<2 mm) and an aneurysm remnant (grade 3) was defined by a residual part of the aneurysmal sac. A digital subtraction angiography control examination was performed immediately after endovascular treatment as a baseline. Angiographic follow-up was performed with DSA. Patients' clinical outcomes were measured by modified Rankin Scale (mRS) score at follow-up visits or by a telephone interview. The follow-up was routinely managed at 6 months, 1 year, and 2 years after the initial treatment.

### Statistical Analysis

Normally distributed continuous data are presented as mean ± SD and categorical data as frequency and percentage. Analysis was carried out with the independent-samples *t*-test and the χ^2^ test. Univariate logistic regression was used to analyze potential factors. Covariates with a univariate *p*-value of <0.2 were included in multivariate Cox proportional hazard regression to identify independent predictors of recurrence. The odds ratio (OR), 95% confidence interval, and *p*-value were determined for factors of the univariate and multivariate models. Statistical significance was defined as *p* < 0.05 and an OR with 95% confidence interval. Statistical analysis was performed using SPSS version 17.0.

## Results

### Clinical Characteristics

The mean age of the 504 patients (219 males and 285 females) was 52.5 ± 10.7 years (Table 1). Of the 558 aneurysms, 228 (40.9%) were ruptured and 330 (59.1%) were unruptured. A total of 94 (16.8%) aneurysms were an irregular shape. A total of 260 (46.6%) aneurysms were treated by coiling alone and 298 (53.4%) aneurysms by stent-assisted coiling. A total of 405 (72.6%) of the 558 aneurysms were located in the anterior circulation and 153 (27.4%) in the posterior circulation. The location of the aneurysm was not associated with recurrence (*p* = 0.907). There were no significant differences in the patients' age, sex, aneurysm shape, and potential risk factors between the two groups (Table 2).

**Table 1:** Clinical characteristics.

**Variables**	***N***
Number of patients	504
**Gender**	
Male	219 (43.5%)
Female	285 (56.5%)
Age (y)	52.5 ± 10.7
**mRS (pre-operation)**	
0–1	396 (78.6%)
2–4	103 (20.4%)
5	5 (1.0%)
**mRS (last follow-up)**	
0–1	443 (87.9%)
2–4	53 (10.5%)
5–6	8 (1.6%)
Last follow-up period (m)	13.6 ± 4.5

**Table 2:** Risk factors for angiographic recurrence after endovascular coil embolization in intracranial saccular aneurysm (*n* = 558).

**Variables**	**Group I (*n* = 501)**	**Group II (*n* = 57)**	***P*-value**
Age (y)	52.62 ± 10.64	51.86 ± 10.73	0.608
Gender (%)			0.846
Male	213 (42.5)	25 (43.9)	
Female	288 (57.5)	32 (57.3)	
Rupture status (%)			<0.001
Yes	192 (38.3)	36 (63.2)	
No	309 (61.7)	21 (36.8)	
Potential risk factors (%)			0.907
<2	364 (72.7)	41 (71.9)	
≥2	137 (27.3)	16 (28.1)	
Therapy (%)			0.008
Non-SAC	224 (44.7)	36 (63.2)	
SAC	277 (55.3)	21 (36.8)	
Location (%)			0.907
AC	364 (72.7)	41 (71.9)	
PC	137 (27.3)	16 (28.1)	
Shape (%)			0.602
Regular	418 (83.4)	46 (80.7)	
Irregular	83 (16.6)	11 (19.3)	
Raymond scale (%)			0.040
1	330 (65.9)	32 (56.1)	
2	144 (28.7)	14 (24.6)	
3	27 (5.4)	11 (19.3)	
Packing density (%)	20.83 ± 3.40	17.26 ± 2.02	<0.001
Aneurysm size (mm)	7.33 ± 5.59	9.62 ± 6.48	0.028

*SAC, stent-assisted coiling; AC, anterior circulation; PC, posterior circulation*.

### Periprocedural Complications

Twenty-three patients suffered from perioperative complications and the total complication rate was 4.6%. Of these patients, seven were treated by coils alone and 16 by stent-assisted coiling. At the final follow-up, permanent neurological complications were found in eight patients, accounting for 1.6% of all cases.

Three patients suffered from arterial thrombosis. Symptomatic cerebral infarction occurred in 11 patients. One patient showed onset of carotid cavernous fistula 10 days after the operation. Five patients had intraoperative bleeding. Coil breakage occurred in two patients. One patient had hydrocephalus after endovascular treatment.

### Results of Clinical Follow-Up

All of the 504 patients participated in the clinical follow-up (mean follow-up period: 13.6 ± 4.5 months) (Table 1). At the last follow-up, 443 patients had a favorable outcome (mRS score: 0–1), 53 patients had an mRS score of 2 to 4, and eight patients had an mRS score of 5 or 6.

### Angiographic Outcome

All of the 558 aneurysms underwent angiographic follow-up after a mean interval of 11.42 months (range: 3.1–26.6 months). After careful review, 501 aneurysms were stable and were included in Group I. Fifty-seven aneurysms showed recurrence and were included in Group II. The total recurrence rate was 10.2%. In this study, we achieved initial total occlusion in 362 (64.9%) of cases, residual neck remnants in 158 (28.3%), and an aneurysm remnant in 38 (6.8%) after endovascular treatment (Table 2). The immediate angiographic results were significantly associated with follow-up outcome (*p* = 0.040). The packing density was significantly associated with follow-up outcome (*p* < 0.001).

In Group I, 224 (44.7%) aneurysms were treated by non-stent assisted coiling and 277 (55.3%) aneurysms were treated by stent-assisted coiling. In Group II, 36 (63.2%) aneurysms were treated by non-stent assisted coiling and 21 (36.8%) aneurysms were treated by stent-assisted coiling. Treatment with or without stents showed a significant association with aneurysmal recurrence (*p* = 0.008). Moreover, the mean aneurysm size in Group I was smaller than that in Group II (7.33 ± 5.59 vs. 9.62 ± 6.48 mm, *p* = 0.028). The factor of aneurysm rupture status was also significantly associated with aneurysmal recurrence (*p* < 0.001) (Table 2).

After multivariate Cox proportional hazard regression analysis, the packing density was independently associated with aneurysmal recurrence (OR = 0.697; 95% confidence interval = 0.636–0.764, *p* < 0.001).

### Retreatment for Recurrent Aneurysms

Of the 57 (10.2%) recurrent aneurysms, 48 received endovascular retreatment and nine remained under continual observation. Of the retreatment cases, 19 were treated with stent-assisted coiling, two with stent deployment alone as failure in re-catheterization of the residual sac, and the rest of the cases were treated with coiling alone.

## Discussion

In the present multicenter study, treating intracranial aneurysms via endovascular therapy resulted in a recurrence rate of 10.2% with angiographic follow-up after a mean interval of 11.4 months (range: 3.1–26.6 months). The perioperative complication rate was 4.6%. Embolization of aneurysms with coils or stent-assisted coiling was effective. We also found that the size of aneurysms, therapy, Raymond scale, rupture status of aneurysms, and packing density were significantly associated with angiographic outcome. The packing density was independently associated with aneurysmal recurrence in multivariate Cox proportional hazard regression analysis.

The size of aneurysms is a crucial risk factor for late recurrence because larger sizes may allow more intra-aneurysmal flow into the aneurysm, affecting thrombosis and recanalization rates (11–14). Our study supports these findings. We found a significant difference in the aneurysm size between cured aneurysms and recurrent aneurysms.

The effect of clinical presentation (ruptured status) on recurrence is still unclear. Some studies have shown that recurrence rates are affected by the rupture status (2, 6, 15). This finding was observed in our study and the rupture status was associated with angiographic recurrence. We consider that the condition of a ruptured aneurysm sac could disturb coil distribution and the surgeon might insert less coils to avoid intraoperative rupture caused by coil perforation. In this condition, a lower packing density and disturbed coil distribution contribute to recanalization of the sac.

Intracranial stents, which are porous tubular mesh made of nitinol or other alloys, have been successfully used to treat wide-necked and complex aneurysms (16–18). The main function of stents is to avoid coil herniation. In addition to containment of coils during coil embolization, stents can also change intra-aneurysmal hemodynamics, induce new intimal hyperplasia, and eventually be wrapped into the vessels, remodeling the parent artery (19, 20). Many studies reported that aneurysmal recurrence was significantly lower in stent-assisted coiling compared with non-stent assisted coiling (19, 21, 22). Our study also found that stents could reduce the recurrent rate of aneurysms.

The reported packing density ranges from 8 to 40% (5, 23, 24). Long-term occlusion of intracranial aneurysms is inversely related to coil packing density within the sac, such that a higher packing density results in a lower recurrence rate (25). Packing density remains important because a low density results in a loose coil mass. This allows high residual blood flow within the aneurysm sac, which may promote delayed coil compaction and significant aneurysmal recurrence (26). Our finding that a low packing density was an independent risk factor for aneurysmal recurrence supports these previous studies.

This study has some limitations. This study was a retrospective cohort with a limited number of patients. Furthermore, different interventional materials were used in this research. Finally, a flow diverter was not used in this study due to the lack of angiographic follow-up and the limited number of patients. Further studies with more data from multiple centers are required to verify the conclusions in the present study.

## Conclusions

The size of aneurysms, therapy, Raymond scale, rupture status of aneurysms, and packing density are significantly associated with the angiographic outcome of aneurysms. A low packing density is an independent risk factor for aneurysmal recurrence. Our study shows that endovascular treatment is still effective for these lesions.

## Data Availability Statement

The raw data supporting the conclusions of this article will be made available by the authors, without undue reservation, to any qualified researcher.

## Ethics Statement

The studies involving human participants were reviewed and approved by IRB of Beijing Tiantan Hospital. The patients/participants provided their written informed consent to participate in this study.

## Author Contributions

ZT contributed to the preparation of the manuscript and data collection. ZT, YinZ, and KW contributed to the data collection. JL, YisZ, XZ, HZ, and MY contributed to data analysis and interpretation. KW and XY contributed to the experimental design and manuscript revision. All authors contributed to the article and approved the submitted version.

## Conflict of Interest

The authors declare that the research was conducted in the absence of any commercial or financial relationships that could be construed as a potential conflict of interest.
